# Reported evidence on the effectiveness of mass media interventions in increasing knowledge and use of family planning in low and middle-income countries: a systematic mixed methods review

**DOI:** 10.7189/jogh.09.020420

**Published:** 2019-12

**Authors:** Jacqueline Safieh, Tibor Schuster, Britt McKinnon, Amy Booth, Yves Bergevin

**Affiliations:** 1 Department of Family Medicine, McGill University, Montréal, Quebec, Canada; 2 Centre for Global Child Health, The Hospital for Sick Children, Toronto, Ontario, Canada; 3 Division of Epidemiology, Dalla Lana School of Public Health, University of Toronto, Ontario, Canada; 4 Department of Public Health Sciences, Queen’s University, Kingston, Ontario, Canada

## Abstract

**Background:**

An estimated 200 million women and girls in low and middle-income countries (LMICs) wish to delay, space or avoid becoming pregnant, yet are not using contraceptives. This study seeks to investigate the effectiveness of mass media interventions for increasing knowledge and use of contraceptives, and to identify barriers to program implementation.

**Methods:**

Using a mixed-methods systematic approach, we searched five electronic databases using pre-determined search strategies and hand-searching of articles of any study design published from 1994 to 2017 of mass media interventions for family planning education. Two reviewers independently applied clearly defined eligibility criteria to the search results, quality appraisal, data extraction from published reports, and data analysis (using meta-analysis and thematic analysis) following PRISMA guidelines.

**Results:**

We identified 59 eligible studies. Although the majority of studies suggest a positive association between media interventions and family planning outcomes, the pooled results are still consistent with possibly null intervention effects. The reported prevalence ratios (PR) for media interventions association with increased contraceptive knowledge range from 0.97 to 1.41, while the PRs for contraceptive use range from 0.54 to 3.23. The qualitative analysis indicates that there are barriers to contraceptive uptake at the level of individual knowledge (including demographic factors and preconceived notions), access (including issues relating to mobility and financing), and programming (including lack of participatory approaches).

**Conclusions:**

There is a need for rigorous impact evaluation, including randomised controlled trials, of mass media interventions on knowledge and uptake of family planning in LMIC settings. Interventions should be better tailored to cultural and socio-demographic characteristics of the target populations, while access to resources should continue to remain a priority and be improved, where possible.

Today, there is an urgent situation facing over “200 million women and girls in developing countries who want to delay, space or avoid becoming pregnant,” yet who “are not using effective methods of contraception” [1,2]. As stipulated at the International Conference on Population and Development in 1994 [1-3], it is imperative that reproductive health programs follow a human rights-based approach, the necessity of cultivating informed decisions, and choices regarding child-bearing. As such, global partners gathered at the 2012 London Summit on Family Planning to launch a “ground-breaking effort to make affordable, lifesaving contraceptives, information, services, and supplies available to an additional 120 million women and girls in the world’s poorest countries by 2020” [2]. Although there has been a notable acceleration of progress in terms of expansion of family planning services in developing countries, there is a critical need to improve knowledge, perceptions and use of contraceptives.

Literature to date has indicated that various factors are linked to individuals’ access to family planning and contraceptives, most significant of which includes: education, poverty and gender inequality [4-8]. Seminal works such as Belaid et al.’s systematic review on demand generation for family planning [9] and Shen and Han’s on entertainment education for health communication [10] each provide critical guidance for health workers seeking to increase health education and autonomy through mass media-scale approaches. A recent study using data obtained from the Demographic and Health Surveys (DHS) Program to analyse the association between mass media exposure and contraceptive use in sub-Saharan Africa found that individuals exposed to mass media communication regarding family planning had 1.93 times the odds of using contraception than those who were not exposed to mass media messages (95% confidence interval (CI) = 0.75, 2.14) [11]. Randomised controlled trials (RCTs) have been conducted to measure the impact between mass-media interventions and health education and outcomes (eg,, HIV testing, child survival) [12-17], yet none have specifically focused on outcomes specific to family planning and contraception.

Therefore, the purpose of systematic review is to synthesise existing evidence on the use of large scale media interventions to increase knowledge and use of family planning and contraception in low & middle-income countries (LMICs). This review uses a mixed methods approach to investigate: a) the quality and limitations of the research being conducted; b) the factors associated with the success of various mass media interventions; and c) the reported evidence on the effectiveness of mass media interventions at increasing knowledge and use of family planning and contraception.

## METHODS

A parallel-results convergent synthesis mixed methods design [18,19] was employed to collect, analyse, and integrate both quantitative and qualitative data reported in the literature.

### Search strategy and selection criteria

Throughout January 2017, searches of five electronic databases were conducted (by JS and AB) using pre-determined search strategies. The search strategy was composed of an arrangement of terms linking concepts of mass media and family planning/contraception (Table S1 in **Online Supplementary Document**). The databases that were searched include: MEDLINE, AMED (Allied and Complementary Medicine), Embase, Global Health, and Social Work Abstracts. The references of articles that matched the eligibility criteria and grey literature, including the websites of relevant organizations (Table S2 in **Online Supplementary Document**), were further searched and were subject to the same screening and selection process.

The systematic review included any social or media-oriented methods of education surrounding contraception and family planning, such as: media messaging (ie, commercial and social marketing), radio and television serial dramas, folk theatre, internet campaigns, text messaging, etc. Studies of all design types were eligible for inclusion if they were published in either English or French from 1994 onwards, presented primary data, the primary focus was on family planning and/or contraception, did not involve new drug testing, differentiated types of media sources, and did not primarily focus on education of sexually transmitted infections (STIs). Studies were excluded if they were testing contraceptive drug development, studied diseases and illnesses (including STIs and/or HIV/AIDS), did not primarily focus on family planning and contraception outcomes, did not differentiate between media sources or types, or focused on non-scalable and therefore irreproducible methods (ie, communication between family members and friends).

### Data analysis and extraction

Following the PRISMA (Preferred Reporting Items for Systematic Reviews and Meta-Analyses) [20] guidelines, after the inclusion and exclusion criteria were developed, two authors (JS and AB) applied the criteria to all search results to determine which articles were eligible for inclusion in this review. First, the titles and abstracts were screened, followed by full paper screening. Any disagreements were resolved through discussion and consensus. All data was analysed for risk of bias and methodological quality assurance using appropriate quality assessment tools, including: TREND Statement for Quasi-Experimental studies, STROBE Checklist for Cross-Sectional studies and Joanne Briggs Institute for Qualitative studies [21-23]. One author (JS) assessed all articles using this tool, and a second author (AB) verified the accuracy of the critical appraisals. Data was extracted from the articles using pre-determined extraction forms. The quantitative data extracted included: population exposure to mass media campaign, knowledge of contraceptives and use of contraceptives. The qualitative data included thematic codes of the results and discussion sections of all included studies [24].

### Synthesis

#### Quantitative synthesis

Quantitative studies were synthesised, where applicable, according to two outcome variables (contraceptive knowledge and contraceptive use) utilizing meta-analysis. Meta-analyses were conducted by study design, separating cross-sectional studies, pre-post studies (where outcomes were measured in the same population before and after an intervention), and control pre-post studies (which contained a control and intervention group that were analysed over time). Meta-analyses were also conducted by key study variables (year, country, intervention, etc.), and visually inspected to determine if any variable appeared to be strongly associated with any of the two outcome variables. To describe the heterogeneity of the intervention effects reported in the literature, we present the range of point estimates (ie, prevalence ratios) across all included studies for each outcome variable. However, instead of computing pooled effect estimates and associated confidence intervals, we reported 95% prediction intervals for the prevalence ratios. This was due to the substantial heterogeneity between the studies, including differences in target populations, methods for dissemination and confounding control. A prediction interval estimates a pre-specified range (eg, the central 95%) of expected effects in a large set of future studies conducted in the same underlying population [25]. To explore the potential role of unmeasured confounding, we present contour plots that depict the minimum required level of confounder imbalance across intervention groups vs the minimum required confounder strength (causal effect on the outcome) that may explain the same estimated range of effects that was observed across the included studies. This graphical approach is based on established bias formulas [26] and previously proposed confounding function methods [27,28].

#### Qualitative synthesis

All studies (qualitative and quantitative) were interpreted (“coded”) by two reviewers (JS and AB). Codes were grouped into themes using inductive and deductive approaches to better understand the barriers to effective programming on reproductive health education through mass media. The theory development utilised a combination of two theories to describe how: a) media messages are translated into knowledge, through the concept of ‘media literacy’ [29], and b) knowledge is subsequently translated into action [30]. Preliminary synthesis consisted of extracting the descriptive characteristics of the studies in a table and producing a textual summary of the results (Tables S2 and S3 in **Online Supplementary Document**). Thematic analysis was then used to extract codes that were then grouped into three main themes [24].

## RESULTS

**Figure 1:**
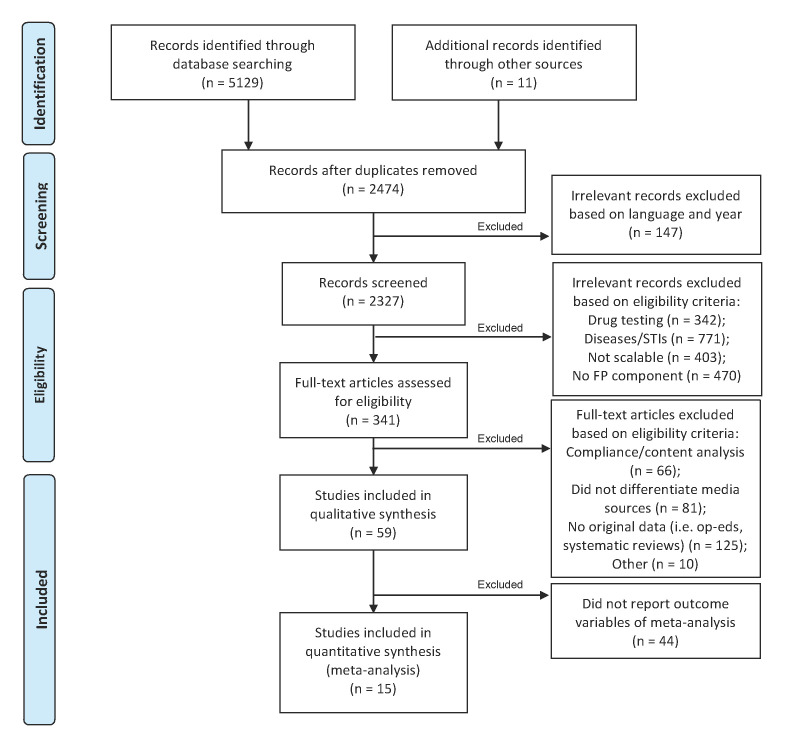
Flowchart of selection of eligible articles

The searches of the databases yielded 5129 citations after duplicates were removed. Exclusions were made to the data set in three stages (**Figure 1**). After the inclusion criteria were applied to the articles, a total of 59 articles from the electronic search of databases were included in the final analysis. An additional six non-peer-reviewed sources were identified through searches of websites and references of included studies. All studies were based on retrospective participant-reported or qualitative data, with pre-post (n = 17), cross-sectional (n = 36) and qualitative (n = 5) study designs. There was one mixed methods study, which combined a cross-sectional survey with a qualitative study design. While randomised controlled trials have recently been initiated within the field of media and reproductive health education (12-17), results either have yet to be published or did not discuss contraception and therefore were not included in the study. After applying appropriate quality appraisal tools [21-23], the methodological quality of studies varied, with the majority categorised as “moderate” overall evidence quality (n = 39) and approximately one third of studies as “high” quality (n = 20) (Table S2 in **Online Supplementary Document**).

The majority of studies were conducted in Africa (n = 28; 16 of which were in Eastern Africa and 7 in Western Africa), followed by Asia (n = 25; 20 of which were in Southern Asia), South America (n = 2), and 4 multi-continental studies. About half the included studies analysed the impact specific programming (n = 7 radio or TV interventions, n = 6 marketing (commercial and social) interventions, n = 2 mobile phones interventions, and n = 16 other forms of mass media programming interventions), while 28 studied the impact of regular (pre-existing, non-content specific) mass media (Table S3 in **Online Supplementary Document**).

### Quantitative results

**Figure 2:**
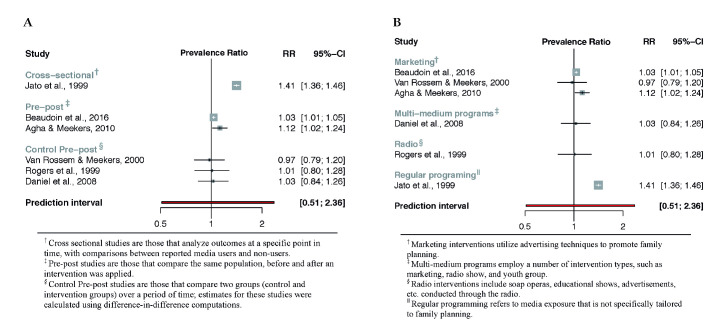
Association between media programs on reproductive health and contraceptive knowledge. **Panel A**. By study design. **Panel B**. By intervention.

**Figure 3:**
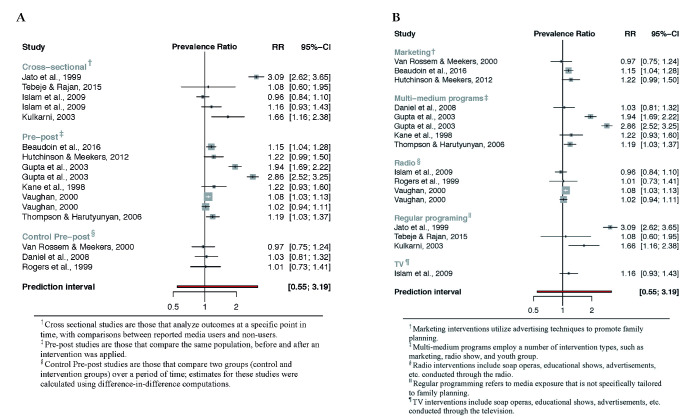
Association between media programs on reproductive health and contraceptive use. **Panel A**. By study design. **Panel B**. By intervention.

The meta-analysis included 15 studies in total and analysed the association between media programming on family planning and two outcome variables: a) knowledge of contraception (n = 5) and b) use of contraception (n = 13) (see **Figure 2** and **Figure 3**). All generated prediction intervals indicate that relevant positive or negative associations may exist between media programs and outcome variables. However, the range of point estimates (ie, prevalence ratios) for included studies for both contraceptive knowledge and contraceptive use suggest either positive or null associations.

**Figure 4:**
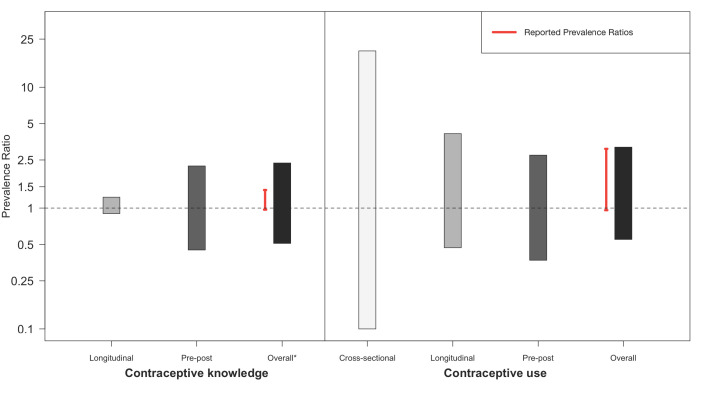
Pooled prediction intervals for prevalence ratios of exposure, knowledge and use due to media interventions by study design. *The overall prevalence ratio prediction interval for contraceptive knowledge included one additional cross-sectional study, for which a prediction interval could not be generated.

The reported prevalence ratios (PR) for contraceptive knowledge range from 0.97 to 1.41, while the 95% prediction interval (PI) ranges from 0.51 to 2.36 (**Figure 4**). There were not enough studies analyzing the relationship between media interventions and contraceptive knowledge to make any conclusions regarding differences between studies or between types of media interventions.

The estimated PRs for contraceptive use yield a similarly wide prediction interval, ranging from 0.54 to 3.23 (**Figure 4**). However, there was substantial heterogeneity in the range of prevalence ratios, with individual studies finding no association to strong positive associations between media interventions and contraceptive use (0.96, 3.06). Control pre-post studies, which represent the highest quality of evidence currently available, tend to demonstrate the weakest association, with a reported point estimate range between 0.97 and 1.01, and a 95% prediction interval between 0.37 and 2.74. When analysed according to type of media intervention, multi-media programs (ie, combining a number of intervention types, such as marketing, radio show, and youth group), and regular ongoing programming (ie, not specifically tailored to family planning) appear to have a stronger association with contraceptive use. The PR point estimate range for each respectively is 1.03 to 2.86 (95% PI = 0.33, 7.30), and 1.08 to 3.09 (95% PI = not estimable). Radio programming showed no association with contraceptive use, with reported point estimates ranging from 0.96 to 1.08 (95% PI = 0.90, 1.21).

The contour plot displaying possible confounder characteristics explaining the range of effects that are consistent with the prevalence ratios reported by the individual studies indicates moderate to extreme confounding scenarios that may be underlying the observed data. For instance, Hutchinson and Meekers 2012 reported a prevalence ratio (risk ratio) for contraceptive use of 1.22 based on a pre-post intervention design. According to the contour plot, an unmeasured confounder that was 4 times more prevalent in the intervention group than in the control group, and, at the same time, was linked with a 1.33-fold increase of prevalence of contraceptive use, may alternatively explain this observed relative effect of 1.22 (Figure S1 in **Online Supplementary Document**).

### Qualitative results

The codes that emerged from deductive/inductive thematic analysis were grouped into three themes, outlined below and summarised in **Table 1** (and Figure S2 in **Online Supplementary Document**).

**Table 1:** Themes emerging from qualitative coding and thematic analysis

Main categories of analysis	# of studies
**Barriers to FP/contraceptive knowledge**	
**Demographic factors:**	
Factors such as education, socioeconomic status, geographic location, marital status, access to media, religion, etc., have been analysed as effecting contraceptive knowledge and use	49
**Innovative methods of distribution of SRH information:**	
Articles discussed the use of text messaging to allow participants to ask questions, working closely with religious leaders, soap operas/serial dramas to address a wide range of social issues, traditional folk events, participatory community-level approaches and the use of social marketing	16
Preconceived notions of Sexual & Reproductive Health (SRH)/Family Planning (FP):	
Western influence/hyper-sexualisation in media; belief that FP is a method of population control; journalists problematizing paradigms of ‘behaviour change’ methods; prior use of traditional/natural methods; fear of side effects	7
**Urban vs rural:**	
Geographic context has a large influence on social networking (organised networks tend to be less present in rural regions) and media methods (while TV is effective, it is not readily available in some rural areas)	3
**Barriers to FP/contraceptive use**	
**Community outreach/networking tends to lead to greater use:**	
Individual outreach (ie, via health care workers) is important (but not scalable); involvement male partners and families in FP decision making is significant, social networking (organised youth groups or non-organised)	18
Lack of agency/self-determination affects health:	
Lack of agency relating to sexual and reproductive health (SRH), financial autonomy and mobility	9
**Barriers to FP/contraceptive programming:**	
Relationship to other SRH factors (ie, STIs/STDs, gender equity, etc.):	
Tackling family planning and contraception in conjunction with other SRH issues tends to be effective and important pre-requisites for social change	10
Top-down vs Bottom-up community-level approaches:	
Articles highlighted the difference between top-down approaches (emphasise use of mass media, social marketing, entertainment education to encourage/model desired behaviours) and bottom-up approaches (emphasise participatory approaches & strengthening capacity of stakeholders)	9

FP – family planning, STI – sexually transmitted infection, STD – sexually transmitted disease

#### Theme 1: Barriers to knowledge

Most studies discussed the influence of various demographic factors, such as education, socioeconomic status, geographic location, marital status, access to media, and religion, on contraceptive knowledge and use [9,10,31-89]. In particular, individuals with a higher level of education, higher socioeconomic status, and who live in urban areas are at an increased likelihood of exposure to family planning messages [53,54,68,70]. Other factors, such as marital status, exposure to media, and religion can influence knowledge in a diverse set of ways, depending on the messages received from spouses, media and religious leaders [47,55,59,65,70,71,74,82]. Moreover, exposure to family planning and contraceptive messages does not necessarily lead to understandings of the various methods available, alluding to the continued importance of education by trained workers [9,44,72,75,79].

Geographic context appears to have a large influence on reproductive health knowledge, vis-à-vis program coverage and social networking. Studies found that there are fewer mainstream media methods (particularly TV) and organised groups in rural regions, resulting in the creation of informal networks to facilitate greater understanding of reproductive health [73,83]. Moreover, while some studies have shown that TV can contribute to increasing family planning education, it is not a preferred method of communication in many rural areas due to lack of availability [38,45,53,76,86-88]. Mobile phones are emerging as a potentially efficient means to reach rural communities [38].

Preconceived impressions about contraceptives, including through previous Western influence and colonialism, hyper-sexualisation in media, the belief that family planning is a method of population control, use of/belief in traditional methods of contraception, and fear of side effects, present other barriers to knowledge and use [34,36,47,48,58,60,67,70,80,89].

Innovative methods of distribution of reproductive health information were mentioned as a facilitator to increased knowledge, highlighting the importance of a diverse strategy to address this issue. Some of the key innovative strategies that were employed by the studies include: text messaging to allow questions [9,37,44,64,90], working with community health workers and religious leaders [47,59,71,82], soap operas/serial dramas [9,10,32,39,41,45,46,49,50,52,56,59,61,65,69,71,74,79,81,85], marketing (social and commercial) [31,32,41,45,52,84], traditional folk events [9,33,55,59,73], and participatory community-building approaches [9,33,44,49,55,61,73,84]. Moreover, some studies argued that combining different innovative methods through ‘complementary messages’ (messages through different sources and medium) “may help to create an environment where the practice of contraception is perceived as a social norm” [10,55,79].

#### Theme 2: Barriers to use

Family planning and contraceptive behaviours appear to be closely related to issues of self-determination and agency, or the ability of a person ability to think, act, and conduct themselves according to their ethical-political values and traditions, particularly for women [9,35,47,58,66,68,72,74,80]. Reduced sexual and reproductive health agency can include fear of discussing potentially sensitive topics with a spouse, inability to obtain contraceptives, spousal/familial refusal to adopt family planning, or issues of financial autonomy and mobility [9,35,47,58,66,68,72,74,80].

Studies highlighted the importance of involving communities in programming, including community outreach and social networking approaches [9,34,35,41,43-45,50,52,53,58,60-62,66,68,72-75,80,82,83,89]. Examples of community involvement include involving male partners and families in family planning decision-making [65,70], social networking [35,41], and organised or non-organised youth groups [35,39,69,78]. The power of social networking to spread information through indirect exposure may also have positive impact on contraceptive awareness [43,83].

#### Theme 3: Barriers to effective programming

Because the issue of family planning is closely related to other sexual and reproductive health issues, many studies have found that tackling contraceptive education in conjunction with other sexual and reproductive health issues tends to be effective [32,41,48,51,54,56,60,62,67,70,74-77,82,84-88]. Some issues that tend to be targeted in conjunction with contraceptive education include STIs (in particular HIV/AIDS), gender equity, and primary and secondary education for girls and boys.

Finally, some studies addressed the difference between top-down approaches, including use of mass media, social marketing, entertainment education to model desired behaviours, and bottom-up approaches, including participatory approaches, strengthening the capacity of stakeholders, and allowing communities to lead in decision-making processes [34,36,47,48,58,60,67,68,70,80,88].

## DISCUSSION

The objective of this study is to better understand the effectiveness of mass media interventions for increasing knowledge and use of contraceptives, including strengths and weaknesses of current programming, in order to guide prospective research and programming on family planning and contraception education. Based on the reported data in the included literature of this review, there appears to be associational evidence that media interventions may be effective at improving population-level outcomes relating to family planning and contraception [52,74,79]. In fact, the vast majority of observed associations suggest positive effects, however, with varying degrees of estimate precision and conclusiveness. Nevertheless, after aggregating the numerical information using inferential statistics ie, 95% prediction intervals, the overall evidence remains inconclusive in answering whether or not mass media interventions have a relevant impact on increased knowledge or use of contraceptives. The range of reported effects (prevalence ratios) for the association between media exposure and contraceptive knowledge varies from 0.97 to 1.41 (95% PI = 0.51, 2.36), while the range of reported effects for the association between media exposure and contraceptive use is from 0.96 to 3.09 (95% PI = 0.55, 3.19) (**Figure 4**). This large range of expected associations in future studies is primarily due to the limited quality of evidence available, ie, lack of rigorous study designs (eg, RCTs) and sophisticated methods for confounding control.

The results of the qualitative analysis may help shed some light on some of these potential factors. The findings indicate that there are barriers to contraceptive uptake at the level of individual knowledge (including demographic factors and preconceived notions), access (including issues relating to agency), and programming (including lack of participatory approaches). These socio-cultural factors be should accounted for when designing media interventions, in order to increase their success.

While great effort was extended to extract as much information as possible from the currently available evidence, we find that this topic is severely under-researched. Because there have not been any rigorous RCTs published on this topic, the review was limited to qualitative, cross-sectional and pre-post studies, meaning that it is not possible to infer causal relationships between exposure to mass media and contraceptive use. Moreover, within the meta-analysis, there was a great deal of heterogeneity between studies and high potential for confounding, as the studies were conducted with differences in years, continents/geography and the program interventions, all of which are factors that could influence the outcome variables. Another limitation based on the available study designs was the issue of contamination. An analysis of exposure to programs among those in the intervention and control group found that many participants in the control group were also exposed to the programs, which may have the effect of minimizing the observed magnitude of association between intervention and outcome. Finally, within the meta-analysis outcome variables, there is no indication regarding the depth of contraceptive knowledge (ie, are participants aware the diversity of options available and of side effects), nor specifics about what methods of contraception individuals have chosen to use and why. Overall, the results provide insights for program managers regarding what steps in the behavior change pathway might require more attention and outline factors to consider when designing and implementing programs.

Although the majority of studies suggest a positive association between media interventions and family planning outcomes, the pooled results (ie, prediction intervals) are still consistent with possibly irrelevant or null intervention effects. Despite these inclusive findings, we cannot rule out the possibility that media interventions are truly having a positive effect on family planning outcomes. In other words, absence of evidence of an effect does not imply evidence of absence of an effect. One inevitable challenge that any review in this field of inquiry faces, is the heterogeneity of study populations and the fact that interventions are typically tailored to the context where they are implemented. It is essential that more research be conducted in order to understand why individuals do not use certain modern methods of contraception, despite increased knowledge and a desire to space, limit or avoid pregnancy.

Future research studies should therefore employ rigorous mixed methods designs, incorporating pragmatic (cluster) randomized controlled trials, to evaluate the impact of various mass media education approaches to improve contraceptive knowledge and use, across heterogeneous populations.

## Additional material

Online Supplementary Document
