# Supplementary material for: Reported evidence on the effectiveness of mass media interventions in increasing knowledge and use of family planning in low and middle-income countries: a systematic mixed methods review
Source: J Glob Health. 2019 Dec 30;9(2):020420. doi: 10.7189/jogh.09.020420 (PMC7686646; doi:10.7189/jogh.09.020420)
Supplement: Online Supplementary Document [file jogh-09-020420-s001.pdf]

Table S1. MEDLINE Search Strategy combining contraceptive and mass media interventions

[Ovid MEDLINE(R) and Epub Ahead of Print, In-Process & Other Non-Indexed Citations, Ovid MEDLINE(R) Daily 1946 to Present]
1. exp Contraception/<br>2. Contraception Behavior/<br>3. Reproductive Health/<br>4. Family Planning Services/<br>5. contracept*.mp.<br>6. "family planning".mp.<br>7. reproductive health.mp.<br>8. 1 or 2 or 3 or 4 or 5 or 6 or 7
9. edutainment.mp.<br>10. mass media/ or radio/ or television/<br>11. infotainment.mp.<br>12. info tainment.mp.<br>13. enter educat*.mp.<br>14. "entertainment education".mp.<br>15. edu tainment.mp.<br>16. radio.mp.<br>17. comic book*.mp.<br>18. storytell*.mp.<br>19. (television or tv).mp.<br>20. folk theatre.mp.<br>21. film.mp.<br>22. cell phone*.mp.<br>23. (text messag* and educat*).mp.<br>24. soap opera*.mp.<br>25. Access to Information/<br>26. Advertising as Topic/<br>27. Answering Services/<br>28. Health Communication/<br>29. Information Seeking Behavior/<br>30. communication intervention*.mp.<br>31. 9 or 10 or 11 or 12 or 13 or 14 or 15 or 16 or 17 or 18 or 19 or 20 or 21 or 22 or 23 or 24 or 25 or 26 or 27 or 28 or 29 or 30
32. 8 and 31

Table S2. Websites screened during data collection for systematic review

Websites screened by hand
• Studies Family Planning<br>• World Health Organization (WHO) Library Database (WHOLIS)<br>• United Nations Population Fund (UNFPA) Library Database<br>• United States Agency for International Development (USAID) Library, and other government development websites<br>• Demographic & Health Survey (DHS) Library

Table S3. Quality appraisal of studies included in systematic review by study design

**Quasi-Experiment**<br>**(Author, yr)** (N=17)	**Questions (TREND for quasi-experimental research)**																				**TOTAL/22**	**Quality**
	1	2	3	4	5	6	7	8	10	11	12	13	14	15	16	17	18	20	21	22		
Agha & Meekers, 2010	1	1	1	1	1	1	0	0	1	1	1	1	1	1	0	1	1	1	0	1	16	Medium
Babalola et al., 2001	1	1	1	1	0	1	1	1	1	1	1	1	1	1	1	1	1	1	1	1	19	High
Beaudoin et al., 2016	1	1	1	1	1	1	0	1	1	1	1	0	0	1	1	1	1	1	0	1	16	Medium
Braun et al., 2016	1	1	1	1	1	1	0	0	0	0	1	0	1	0	0	1	1	1	0	1	12	Medium
Daniel et al., 2008	1	1	1	1	1	1	0	1	1	1	1	0	0	1	1	1	1	1	0	1	16	Medium
Gupta et al., 2003	0	1	1	0	1	1	1	0	1	1	1	1	1	1	0	1	1	1	0	1	15	Medium
Hutchinson & Meekers, 2012	1	1	1	1	1	1	1	0	1	1	1	0	1	1	0	1	1	1	1	1	17	High
Jato et al., 1999	1	1	1	1	1	1	0	0	0	1	1	1	0	0	0	1	1	1	0	1	13	Medium
Kane et al., 1998	1	1	0	1	0	0	0	0	1	0	0	1	1	1	1	1	1	1	1	1	13	Medium
Kim et al., 2007	1	1	1	1	1	1	1	0	1	1	1	1	1	1	1	1	1	1	1	1	19	High
L'Engle et al., 2013	1	1	0	1	1	1	0	0	1	1	1	1	1	0	1	1	0	1	1	1	15	Medium
Rogers et al., 1999	1	1	0	1	0	0	1	1	1	1	1	1	1	1	1	1	1	1	0	1	16	Medium
Sharma et al., 2011	1	1	0	0	0	1	1	1	1	1	1	1	1	1	1	1	1	1	0	1	16	Medium
Speizer et al., 2014	1	1	0	1	1	1	1	1	0	1	1	1	1	0	1	1	1	1	0	1	16	Medium
Underwood & Kamhawi 2014	1	1	1	1	1	1	0	0	1	1	0	1	1	1	1	1	1	1	0	1	16	Medium
Van Rossem & Meekers, 2000	1	1	1	1	0	1	0	1	1	1	1	1	0	1	0	1	1	1	0	1	15	Medium
Vaughan, 2000	1	1	1	1	0	1	1	0	0	1	0	1	1	1	0	1	1	1	0	1	14	Medium

For all questions l = yes; 0 = no; Publications with a score < 10 were excluded; 11 – 16 = Medium; 17+ = High

**Cross sectional (Author, yr)** (N=37)	**Questions (Strobe for cross-sectional research)**																						**TOTAL/22**	**Quality**
	1	2	3	4	5	6	7	8	9	10	11	12	13	14	15	16	17	18	19	20	21	22		
Agha & van Rossem, 2002	0	1	1	1	1	1	1	1	0	0	1	1	1	0	1	0	1	1	0	1	1	1	16	Medium
Ajaero et al., 2016	1	1	1	1	1	1	0	1	1	1	1	1	1	1	1	1	1	1	0	1	0	0	18	High
Akufuah & Sossou, 2008	0	1	1	1	1	0	1	1	0	0	0	1	1	1	1	1	1	1	0	1	0	0	14	Medium
Alemayehu et al., 2016	0	1	1	1	1	1	1	0	0	1	1	1	0	1	1	1	0	1	0	1	0	0	14	Medium
Arora et al., 2013	1	1	1	1	0	1	1	0	0	1	1	1	1	1	1	1	1	1	0	1	0	1	17	Medium
Bajoga et al., 2015	1	1	1	1	1	1	1	1	0	1	1	1	1	1	1	1	1	1	1	1	1	1	21	High
Banerjee et al., 2015	1	1	1	1	1	0	1	1	0	0	1	1	1	1	1	1	1	1	1	1	1	0	18	High
Bankole, 1996	1	1	1	1	1	1	1	1	0	1	1	1	1	1	1	0	0	1	0	1	0	0	16	Medium
Barker et al., 2013	1	1	1	1	1	0	0	1	0	0	1	1	1	0	1	1	0	1	1	1	0	0	14	Medium
Boulay et al., 2002	1	1	1	1	1	1	1	1	0	1	0	0	1	1	1	0	1	1	0	1	1	1	17	Medium
Char et al., 2011	1	1	1	1	1	1	1	1	0	1	0	0	1	1	1	0	1	1	0	1	1	1	17	Medium
de Oliveira et al., 2014	1	1	1	1	1	1	1	1	1	1	1	1	1	1	1	1	1	1	1	1	0	1	21	High
Dixit et al., 2013	0	1	1	1	1	1	1	1	0	0	1	1	0	1	1	1	0	1	0	1	0	0	14	Medium
Habibov & Zainddinov, 2015	1	1	1	1	1	1	1	1	0	1	0	1	1	1	1	1	1	1	1	1	0	0	18	High
Islam & Kabir, 1998	0	1	1	1	1	0	0	0	0	1	1	1	0	1	1	1	1	1	0	1	0	0	13	Medium
Islam et al., 2009	0	1	1	1	1	1	1	1	0	1	1	1	1	1	1	1	1	1	0	1	1	0	18	High
Jin & Jeong, 2010	1	1	1	1	1	0	1	0	0	0	1	1	0	0	0	1	1	1	1	1	1	0	14	Medium
Kabir & Islam, 2000	0	1	1	1	1	1	1	1	0	1	0	0	0	1	1	1	1	1	0	1	1	0	15	Medium
Koringa et al., 2015	0	0	1	1	1	0	0	1	0	0	1	1	0	1	1	0	1	1	0	1	0	1	12	Medium
Kulkarni, 2003	0	0	1	0	1	1	1	1	0	1	1	1	0	1	1	0	1	1	0	1	0	0	13	Medium
Meekers et al., 2007	1	1	0	1	1	1	1	1	0	0	1	1	0	1	1	1	1	1	1	1	0	1	17	Medium
Melka et al., 2015	1	1	1	1	1	1	1	1	0	1	1	1	1	1	1	1	1	1	1	1	0	1	20	High
Nanavati et al., 2006	0	1	1	1	1	1	0	1	0	1	1	1	0	1	1	0	1	1	0	1	0	1	15	Medium
Nawaz et al., 2012	1	0	1	1	1	1	1	1	0	1	1	1	1	1	1	0	1	1	0	1	0	0	16	Medium
Okeowo & Olujide, 2014	0	1	1	1	1	1	1	1	0	1	1	0	1	1	1	1	1	1	0	1	1	1	18	High
Okigbo et al., 2015	1	1	1	1	1	0	1	1	0	0	1	1	0	1	1	1	1	1	1	1	1	1	18	High
Paek et al., 2008	1	1	1	1	1	0	1	0	0	0	1	1	0	1	1	1	1	1	1	1	1	0	16	Medium
Schwandt et al., 2015	1	1	1	1	1	1	1	1	0	1	1	1	0	1	1	1	1	1	1	1	0	1	19	High
Shrestha et al., 2014	1	1	1	1	1	1	1	1	0	0	0	0	1	1	1	0	0	1	0	1	0	0	13	Medium
Somba et al., 2014	1	1	1	1	1	0	0	1	0	1	1	0	1	1	1	0	0	1	1	1	1	0	15	Medium
Tebeje & Rajan, 2015	1	1	1	1	1	1	0	1	0	1	1	1	0	1	1	1	1	1	0	1	0	1	17	Medium
Thompson & Harutyunyan, 2006	1	1	1	1	1	0	1	1	0	1	1	1	1	1	1	1	1	1	1	1	1	1	20	High

Valente & Poppe, 1996	0	1	1	1	1	1	0	1	0	1	0	0	0	1	1	1	1	1	1	1	0	0	14	Medium
Westoff & Koffman, 2011	1	1	1	1	1	1	1	1	0	1	1	1	1	1	1	1	1	1	0	1	1	0	19	High
Westoff et al., 2011	1	1	1	1	1	1	1	1	0	1	1	1	1	1	1	1	1	1	0	1	1	0	19	High
Westoff, 2012	1	1	1	1	1	1	1	1	0	1	1	1	1	1	1	1	1	1	0	1	1	0	19	High
Wosu, 2013	1	1	1	1	1	0	1	0	0	1	1	1	0	1	1	1	1	1	1	1	0	1	17	Medium

For all questions l = yes; 0 = no; Publications with a score < 12 were excluded; 12 – 18 = Medium; 18+ = High

**Qualitative (Author, yr)** (N=6)	**Questions (JBI for qualitative research)**										**TOTAL/8**	**Quality**
	1	2	3	4	5	6	7	8	9	10		
Barker et al., 2013	1	1	1	1	1	0	0	1	1	1	8	Medium
Dutta & Basnyat, 2008	1	1	1	1	1	1	0	1	1	1	9	High
Kagurusi, 2013	1	1	1	1	1	1	0	1	1	1	9	High
Khan et al., 2008	1	1	1	1	1	0	0	1	1	1	8	Medium
Ochako et al., 2015	1	1	1	1	1	0	0	1	1	1	8	Medium
Paz Soldan, 2004	1	1	1	1	1	1	1	1	1	1	10	High

For all questions l = yes; 0 = no; Publications with a score < 5 were excluded; 6 – 8 = Medium; 9 – 10 = High

Table S4. Characteristics of all studies included in systematic review

Author, Year	Study Design	Setting [Country]	Continent	Dates	Participants	Sample Size	Data Collection	Intervention	Results Summary	Quality Appraisal
Agha & Meekers, 2010	Quasi-Experi.	[Pakistan]	Southern Asia	April-June 2009	Married Men, 15-49	N= 627; Random selection	Cross-sectional survey	Marketing: Media advertisement on condom use	Nearly ½ men surveyed never used a condom with wives, and almost 13% consistently use condom. 15% of men were aware of Touch ad, 15% had unconfirmed awareness of FP ads.	Medium
Agha & van Rossem, 2002	Cross-sectional	[Tanzania]	Eastern Africa	Dec. 1999	Sexually experienced men and women	N= 2712	Survey at shops that sell female condom	Marketing: (radio & newspaper) on use of female condoms	About 6% exposed to peer education, 6% had given an explanation by provider on use of female condom. 38% had been exposed to mass media campaigns promoting female condom. Mass media exposure increased likelihood that a man or woman would discuss use of female condom with partner.	Medium
Ajaero et al., 2016	Cross-sectional	[Nigeria]	Western Africa	2013	Women, 15–49	N=38,948	DHS cross-sectional	Regular family planning education programs (Radio, TV, NP)	Access to mass media messages increases the likelihood of the use of family planning; and people with higher socioeconomic status & those from the Southern part of the country make more use of family planning.'	High
Akufuah & Sossou, 2008	Cross-sectional	[Ghana]	Western Africa	2008	Men, 19-65	N= 200; Non-random and convenient sampling	Demographics survey & face-to-face interviews	Regular family planning education programs (Radio, TV, NP)	Demographic factors such as education, religion, types of marital relationship, and exposure to mass-media education have significant effects on the participants' increased knowledge, changing attitudes, and family planning and reproductive decision-making.	Medium
Alemayehu et al., 2016	Cross-sectional	Afar region [Ethiopia]	Eastern Africa	Jan-13	Women, 15–49	N=602	DHS cross-sectional	Regular family planning education programs (Radio, TV, NP)	The low coverage of family planning in the region could be due to the influence of husband, religious and clan leader. Attitude of women towards family planning methods, possession of radio, monthly income, and edu- cational status were associated with family planning utilization. Health facilities were the main source of information for the respondents 150(40.2 %).'	Medium

Arora et al., 2013	Cross-sectional	Universities in Dehli [India]	Southern Asia	2013	Female students	N=220	Cross-sectional survey	Regular family planning education programs (Radio, TV, NP)	Awareness about emergency contraceptive pills is quite high but knowledge regarding the correct timing and usage is poor, and there is a fear of side effects	Medium
Babalola et al., 2001	Quasi-Experi.	[Cameroon]	Middle Africa	1998-1999	Women	N= 571	1998 DHS and SFPS	CHW: Family planning promotion at delivery sites	More than 1/3 women exposed to Gold Circle campaign, 52% of whom mentioned being exposed through TV. Women with primary or secondary education were 4 and 6 times (respectively) as likely as those who weren't exposed. Exposure associated with significant increase in level of family planning ideation and increased likelihood of using modern contraceptive method (80%).	High
Bajoga et al., 2015	Cross-sectional	Abuja, Ilorin, Kaduna, Zaria, Benin & Ibadan [Nigeria]	Western Africa	2010	Women, 15–24	N=5765	Baseline survey of NURHI	Regular family planning education programs (Radio, TV, NP)	Approximately 71% of our sample was exposed to FP messages in the media within the three months preceding the survey. The main sources of media exposure were mobile phones (48%), radio (37%), and television (29%). Controlling for relevant factors, recent media exposure to FP messages predicted both sexual experience and use of modern contraceptive methods, although there were city-level differences.'	High
Banerjee et al., 2015	Cross-sectional	Jharkhand [India]	Southern Asia	2012	Women, 15-49	N=1381	Cross-sectional survey	Programs: Youth-focused interventions	Lack of knowledge about sexual and reproductive health in this context indicates that young rural Indian women would benefit from a youth-friendly SRH intervention to improve the women's self-efficacy and decision-making capacity regarding their own health. A communication intervention using outreach workers may be a successful method for delivering this intervention.	High
Bankole, 1996	Cross-sectional	[Nigeria]	Western Africa	1990 & 1993	Nigerian women	N=6696; N=1221	DHS 1990; & follow-up interviews	Regular family planning education programs (Radio, TV, NP)	37% of women who heard or seen media messages intended to use contraception in future, compared to only 19% of those who were not exposed to media messages.	Medium

Barker et al., 2013	Cross-sectional & Qualitative	[Rwanda]	Eastern Africa	2007 – 2009	Women, 15-49 at two hospitals and four health centres	N=299	Cross-sectional survey	Radio: Entertainment-education soap opera	The results showed that the radio soap opera led to a reduced mean desired family size among listeners (3.61 to 2.94, p<.001), and listeners were more likely to talk to their spouse about FP, and were more likely to know where to obtain male & female condoms. The qualitative focus groups found that listeners "noted that they had learned from the programme, and had found it entertaining and meaningful on a personal level"	Medium
Beaudoin et al., 2016	Quasi-Experi.	[Pakistan]	Southern Asia	April 2009 – June 2010	Women, 15–49	N=1012	N/A	Marketing: Touch Condom Media Campaign (TV ad for condom brand)	"Effectiveness of the Touch condom media campaign is documented in the beneficial changes in condom-related out-comes across the three waves along with the specification of the effects of confirmed Touch ad recall on these outcomes"	Medium
Boulay et al., 2002	Cross-sectional	Dang District [Nepal]	Southern Asia	Nov-97	Women, 15–49	N=667	Semi-structured interviews	Indirect Exposure: to Radio Communication Project	"While direct exposure to the radio program appeared to in□uence family planning knowledge, indirect exposure was more strongly associated with contraceptive use. These □ndings suggest that program evaluations that ignore indirect exposure un- derestimate the impact of a mass media program on behavior."	Medium
Braun et al., 2016	Quasi-Experi.	Dar se Salaam region [Tanzania]	Eastern Africa	13-Jan	Community health workers & clients	N=25 (CHW); N=175	Semi-structured interviews & surveys	Mobile Phones: Mobile technology to support community health workers	Mobile phones can be effective tools to support CHWs with FP counseling, screening and referrals, data collection and reporting, and communication	Medium
Char et al., 2011	Cross-sectional	Rural regions [India]	Southern Asia	Mar-05 – Sept-05	Unmarried men, 17-22	316 (survey); 38 (interview)	Semi-structured interviews	Marketing: DKT India, contraceptive social marketing campaign	Young unmarried men in rural India are underserved with regard to SRH information and services, because they are not recognized as key targets under the public health system, and they receive their limited knowledge and information mainly from the mass media	Medium

Daniel et al., 2008	Quasi-Experi.	Bihar [India]	Southern Asia	2002-2003; 2004	Women, <25 with no more than 1 child	N=199; N=2080	Semi-structured interviews	Programs: PRACHAR Project (various communication interventions)	"Culturally appropriate, community-based communication programs that target youth and those who influence their decisions can create demand for contraception among young couples and lead to increased contraceptive use."	Medium
de Oliveira et al., 2014	Cross-sectional	[India]	Southern Asia	2005 – 2006	Men & women, 15-54	N=198754; random sampling	National Family Health Survey – in depth interviews	Regular family planning education programs (Radio, TV, NP)	The persistent dominance of sterilization in the Indian family planning programme is largely determined by socioeconomic conditions. Reproductive health programmes should address the socioeconomic barriers and consider multiple cost-effective strategies such as mass media to promote awareness of modern temporary methods.	High
Dixit et al., 2013	Cross-sectional	Slums in Jaipur [India]	Southern Asia	Jun-12 – Oct-12	Married men, 18-49	N=400	Semi-structured interviews	Regular family planning education programs (Radio, TV, NP)	There is a gap in knowledge between attitudes towards family planning between men & women; men should equally participate of efficient results in family planning programs	Medium
Dutta & Basnyat, 2008	Qualitative	[Nepal]	Southern Asia	1995 – 2008	N/A	N/A	Narrative analysis	Radio: Entertainment-education soap opera	"Practitioners adopting the participatory framework for health education projects ought to examine the ways in which the cultural context and the voices of cultural participants are reflected... Projects that have a predetermined agenda and seek to use participatory platforms to diffuse this agenda... are fundamentally top-down projects in which participation is used as another communication tool for achieving predetermined change"	High
Gupta et al., 2003	Quasi-Experi.	[Uganda]	Eastern Africa	1995 – 1999	Women and men living in DISH districts, Women: 15-49, Men: 15-54	N=2316 women; N=663 men	Nationally representative survey	Programs: DISH behavior change communication programs (logo, radio & TV entertainment education, posters)	"Results indicate that exposure to BCC messages was associated with increased contraceptive use and intention to use. Some evidence of self-reported bias is found, and the pathways to fertility-related behavioral change appear different women and men."	Medium

Habibov & Zainddinov, 2015	Cross-sectional	[Kyrgyzstan] & [Tajikistan]	Central Asia	2012-2014	Women, 15–49	N=3041; N=3437	DHS cross-sectional	Regular family planning education programs (Radio, TV, NP)	"Viewing family planning messages on TV improves the chances of using modern contraception for a woman who actually saw the messages by about 11 and 8 per cent in Kyrgyzstan and Tajikistan, respectively...By contrast, the effect of hearing family planning messages on radio is not significant in both countries."	High
Hutchinson & Meekers, 2012	Quasi-Experi.	[Egypt]	North Africa	2004-2005	Men & women in villages	N=2088	2 waves of Menya Village Health Surveys	Marketing: multimedia health communication campaign 'Your Health, Your Wealth'	All of the estimators find positive effects of the 'Your Health, Your Wealth' campaign on reproductive health outcomes, though the magnitudes of those effects diverge, often considerably	High
Islam & Kabir, 1998	Cross-sectional	[Bangladesh]	Southern Asia	1993	Currently married women, under 50	N=3100	In-depth interviews	Regular family planning education programs (Radio, TV, NP)	Age, number of living children, religion, radio ownership, approval of FP are important determinants affecting use of modern contraception. Policy implications in sense that mass media will play an important role in future MCH-FP	Medium
Islam et al., 2009	Cross-sectional	Modhupur & Haluaghat [Bangladesh]	Southern Asia	Dec-07 – Feb-08	Indigenous peoples (Garos), XX	N=223; purposive sampling	Semi-structured interviews	Regular family planning education programs (Radio, TV, NP)	Television was most significant form of mass media, more than radio and newspapers. About 80-6% heard of FP messages through TV, Radio was 55-3%, and Newspapers was 22-7%. Contraceptive prevalence rate is higher (79-5%) in study area than national level (55-8%).	High
Jato et al., 1999	Quasi-Experi.	[Tanzania]	Eastern Africa	1991-1994	General population	N=4225	DHS & Tanzania Knowledge, Attitude & Practice Survey	Regular family planning education programs (Radio, TV, NP)	More types of media women exposed to, more likely they are to practice contraception. Women who listened to 6 media sources of FP messages were 11 times likely to use modern contraception.	Medium
Jin & Jeong, 2010	Cross-sectional	[Korea]	Eastern Asia	2005 – 2007	Men & women, 17-31; college students	N=176	Content analysis & survey	Television: Entertainment-education soap opera	"Results indicated that the perceived prevalence of having fewer children in married life was significantly affected by exposure to dramas which positively feature single life and having fewer children in married life on television."	Medium

Kabir & Islam, 2000	Cross-sectional	[Bangladesh]	Southern Asia	1996-1997	Married women; <50	N=871; random sample	DHS 1996-1997	Regular family planning education programs (Radio, TV, NP)	Mass media family planning messages on the radio are more effective than those on TV, yet the likelihood of using contraception is higher if the women had access to any type of mass media on family planning messages	Medium
Kagurusi, 2013	Qualitative	East Africa	Eastern Africa	2008-2012	Journalists	N=60	Semi-structured interviews & focus groups	Programs: 3 short courses on behaviour change communication for journalists	The barriers to behaviour change communication (BCC) regarding family planning were observed to be insufficient BCC skills, journalists' conflict of interest, interests of media houses, inaccessible sources of family planning information, editorial ideologies and absence of commercially beneficial demand.	High
Kane et al., 1998	Quasi-Experi.	[Mali]	Western Africa	Nov-92 – Aug-93	General population; Men: 15-59, Women: 15-49	N=824; N=868	Surveys	Programs: multimedia FP education (via traditional theatre, music, radio, TV, etc.)	High level of exposure to and agreement with messages. Dramatic drop found in proportion of men and women who believe that Islam opposes family planning. Logistic regression indicate contraceptive knowledge and use and more favourable attitudes toward family planning are positively associated with intensity of exposure.	Medium
Khan et al., 2008	Qualitative	[India]	Southern Asia	2007 – 2008	Youth boys and girls, 13-24	N= 234 boys, 189 girls	Semi-structured interviews	Regular family planning education programs (Radio, TV, NP) -- Effects on community gatekeepers	Parents as well as other gatekeepers are worried about rapid changes in aspiration, expectation, and behaviour of young men. Most of them concerned about increasing drinking habit, use of drugs, and changing values of sexuality leading to various risk behaviours among young men	Medium
Kim et al., 2007	Quasi-Experi.	West Java [Indonesia]	South-Eastern Asia	2003	Married women, 18-49	N=1200; random selection	Population-based household survey	Programs: Smart Patient, Smart Community (waiting room intervention) & Sahabat (health worker training)	Ave. respondents were 32. 40+% in each study were 25-34, majority had 2 children. Large proportion of women in intervention had attended secondary education than in control areas.	High

Koringa et al., 2015	Cross-sectional	Urban slums in Jamnagar, Gujarat [India]	Southern Asia	2013	Women, 15-49	N=450; cluster sampling	Structured interviews	Regular family planning education programs (Radio, TV, NP)	There is a need for constant interaction of health functionaries with the eligible couple for boosting family planning program; strengthening behavior change communication (BCC) activities within health department and nonhealth department; improving knowledge, attitude, and practice in the community toward family planning practices	Medium
Kulkarni, 2003	Cross-sectional	Goa [India]	Southern Asia	1997	Married women, 15-45;	N=250; stratified random sampling	Pre-designed & tested questionnaire	Regular family planning education programs (Radio, TV, NP)	Current use of FP practice by women was 48.4$, use of FP methods found to be positively correlated with women's exposure to information on FP methods in television, radio, and newspapers. Hindus comprised 66.4%, 26.4 were Christians, 13.2% were Illiterates, 11.6% had primary level education, 22% had secondary education, and 53.2% had secondary and more. 79.2% of women had access to television, 46.8% to radio, and 46.3% to newspapers.	Medium
L'Engle et al., 2013	Quasi-Experi.	[Tanzania]	Eastern Africa	2005	Married men with at least 1 child	N=600; representative sample	Cross-sectional survey	Mobile Phones: Automated family planning information	Outreach through mobile phones & text messaging is an effective method of delivering family planning information to younger people, women and men of reproductive age	Medium
Meekers et al., 2007	Cross-sectional	[Malawi]	Eastern Africa	2004	Women, 15–49; Men, 15-54	N=11698; N=3261	DHS cross-sectional	Radio: Programs on family planning & health	"The radio programs were found to have a significant impact on family planning discussion with one's partner. The programs' effect on condom use was limited, however... This limited impact suggests that such radio communication campaigns need to be informed by research identifying the specific constraints to current condom use in Malawi."	Medium

Melka et al., 2015	Cross-sectional	[Ethiopia]	Eastern Africa	Apr-14	Married women, reproductive age	N=1003; multistage sampling	Cross-sectional survey	Regular family planning education programs (Radio, TV, NP)	Efforts need to be aimed at women empowerment, health education, and encouraging open discussion of family planning by couples	High
Nanavati et al., 2006	Cross-sectional	Urban and rural regions [India]	Southern Asia	2005 – 2006	School girls, 15-17	N=200, sample respondents	Cross-sectional questionnaire survey	Regular family planning education programs (Radio, TV, NP)	Television main source of knowledge about contraceptives in both rural and urban areas for school girls, 60% of rural girls had knowledge of availability, only 31% in urban areas. 81% rural girls preferred marriage between 18-20, only 41% of urban girls did. 59% of urban preferred marriage after 21. Many girls didn't approve of premarital sexual relations.	Medium
Nawaz et al., 2012	Cross-sectional	Punjab [India]	Southern Asia	2005	Urban and rural men	N=600; random sampling	Semi-structured interviews	Regular family planning education programs (Radio, TV, NP)	For general acceptance of contraceptive use, men along with women should be include in the target group as most of the decisions regarding family planning and contraception necessitate prior approval of male partners	Medium
Ochako et al., 2015	Qualitative	[Kenya]	Eastern Africa	Apr-12	Sexually active women, 15-24	N=34	Puposive sampling; in-depth interviews	Programs: Youth focussed behaviour change communication campaign	"Findings from this research confirm that awareness and knowledge of contraception do not necessarily translate to use. The main barriers to modern contraceptive uptake among young women are myths and misconceptions. The findings stress the influence of social network approval on the use of family planning, beyond the individual's beliefs. In such settings, family planning programming should engage with the wider community through mass and peer campaign strategies."	Medium
Okeowo & Olujide, 2014	Cross-sectional	Rural regions in Ogun State [Nigeria]	Western Africa	2008	Women, 18-50	N=120	Cross-sectional survey	Regular family planning education programs (Radio, TV, NP)	Reliable sources (health personnel) were being utilised for information on family planning, which gave them adequate knowledge of the subject and consequently led to high utilisation of family planning methods among them, however many misconceptions about contraceptives persist	High

Okigbo et al., 2015	Cross-sectional	[Kenya], [Nigeria], [Senegal]	Eastern & Western Africa	2012-2013	Men, 15-59	N=696; N=2311; N=1613	Cluster representative sampling	Programs: Urban Reproductive Health Initiative (URHI)	"In Kenya, those who participated in URHI-led community events had four times higher odds of reporting use of modern methods (aOR: 3.70; $p < 0.05$) while in Senegal, exposure to URHI-television programs (aOR: 1.40; $p < 0.05$) and having heard a religious leader speak favorably about FP (aOR: 1.72; $p < 0.05$) were associated with modern contraceptive method use. No such associations were observed in Nigeria."	High
Paek et al., 2008	Cross-sectional	[Uganda]	Eastern Africa	2001	Mean age: 31.65, People who live 5-km from a health center	N= 350	Delivery of Improved Services for Health (DISH) Survey	Regular family planning education programs (Radio, TV, NP) -- Effect on FP gender norms	All four variables were significant predictors of family planning behaviour. Interpersonal communication was positively related to family planning behaviour, exposure to health-related radio planning was not significant predictor of dependent variable.	Medium
Paz Soldan, 2004	Qualitative	Mangochi District [Malawi]	Eastern Africa	Mar-02 – Jun-02	Men and women in rural district	N=47	Semi-structured interviews & focus groups	Indirect Exposure: Interpersonal communication on mass media and behaviour change	Women's knowledge was based on conversations with other women, men "knew" about practices from observations of others' family size and child spacing. Main trigger for men and women for FP discussions was gossip. Women generally heard about FP at hospital, men first heard from radio or health-drama group.	High
Rogers et al., 1999	Quasi-Experi.	[Tanzania]	Eastern Africa	1993-1997	General population	N=2750 households	5 annual surveys	Radio: Entertainment-education soap opera	The entertainment-education radio soap opera (Radio Tanzania) had a significant effect on listener's family planning adoption, self-efficacy regarding family planning and influenced listeners to discuss family planning with their spouse	Medium
Schwandt et al., 2015	Cross-sectional	[Nigeria] & [Egypt]	Northern & Western Africa	2011; 2004-2007	Men, 15-59; Women 15-49	N=5551 (N-m); N=16144 (N-w); N=2240 (E-w)	NURHI data & Egyptian longitudinal MVHS data	Regular family planning education programs (Radio, TV, NP) -- Integrated Gateway Model	"The key gateway behavior identified in both datasets was spousal communication about family planning, whereas the key gateway factor was exposure to family planning messages."	High

Sharma et al., 2011	Quasi-Experi.	[Nepal]	Southern Asia	1996, 2001, 2006	Individuals, households, communities & programs	N= 8428; N=8726; N=10793; Nationally representative	Nepal Family Health Survey & DHS	Regular family planning education programs (Radio, TV, NP)	Exposure of women to family planning messages through health facilities, family planning workers, radio, and television increased the odds of using modern contraceptives; impact of family planning information on contraceptive use varied according to ethnicity	Medium
Shrestha et al., 2014	Cross-sectional	Dkulikhel [Nepal]	Southern Asia	2013 – 2014	Couples 15-49	N=515 couples	Survey & follow-up	Regular family planning education programs (Radio, TV, NP)	Education plays a vital role in acceptance of family planning; various media (radio, television) tend to make people aware of methods of family planning	Medium
Somba et al., 2014	Cross-sectional	Universities in Dar se Salaam region [Tanzania]	Eastern Africa	Jun-13 – Oct-13	Women, 19-37	N=253	Cross-sectional survey	Regular family planning education programs (Radio, TV, NP)	Most of the students were sexually active and had knowledge of contraception, however, rate of contraception use is still low. There is a need for advocacy for adolescence reproductive health education to promote the use of the available contraceptive services amongst university students.	Medium
Speizer et al., 2014	Quasi-Experi.	[India, Kenya, Nigeria, Senegal]	Multi-country	2010 – 2013	Women, 15+	N=13075; random sample	DHS & in depth interviews	Programs: Urban Reproductive Health Initiative (URHI)	Targeted, multilevel demand generation activities can make an important contribution to increasing modern contraceptive use in urban areas and could maternal and child health and access to reproductive health	Medium
Tebeje & Rajan, 2015	Cross-sectional	Rural regions in Dugda Woreda [Ethiopia]	Eastern Africa	2012	Couples	N=240; purposive sampling	Semi-structured interviews	Regular family planning education programs (Radio, TV, NP)	Husband's educational status, desire for male child, family size preference, spousal communication, wife participation in decision making, fear of the side effects, mass media exposure, information seeking behavior and couples perception of FP are significant factors in determining contraceptive use	Medium

Thompson & Harutyunyan, 2006	Cross-sectional	[Armenia]	Western Asia	1997	Married couples	N=1212 couples	Cluster representative sampling	Programs: Entertainment Education (Green Path Campaign for Family Health)	"Exposure to the campaign was associated with significant increases in factors associated with contraceptive behavior change: knowledge, favorable attitudes toward modern methods, favorable attitudes toward family planning services, and information seeking and utilization of family planning services."	High
Underwood & Kamhawi 2014	Quasi-Experi.	[Jordan]	Western Asia	2011	Religious leaders & mosque attendees	N=390 RL; N=857 attendees	Baseline & Endline Surveys	Programs: FP education program for religious leaders	Intervention mosque goers who recalled messages were more likely to report taking relevant actions; trained RLs compared with their counterparts were more effective in message dissemination	Medium
Valente & Poppe, 1996	Cross-sectional	[Peru]	South America	1993 - 1994	Peruvian population; 15-45	N=1500; random sampling	Semi-structured interviews	Indirect Exposure: Interpersonal communication on mass media and behaviour change	Those who initiated contraceptive use recently were more likely to discuss messages with physicians than those who never used contraceptives.	Medium
Van Rossem & Meekers, 2000	Quasi-Experi.	[Cameroon]	Middle Africa	Jul-96 – Nov-97	General population	N=1633; cluster sampling	Structured interviews	Marketing: Adolescent reproductive health program	91% of respondents reported hearing program. 5% were familiar with program. 2/3 respondents reported to having form of personal contact with HJ program, 28% were actively involved, only 60% talked to HJ member, 47% attended at least 1 meeting	Medium
Vaughan, 2000	Quasi-Experi.	[St. Lucia]	South America	Jan-95 – Sep-98	General population	N=1238	Semi-structured interviews	Radio: Entertainment-education soap opera	35% had listened to Apwe Plezi, 12% who listened 1/week. 16% knew a slang word for condoms, non-listeners were more likely to trust FP workers and considered having lower number of children. 14% listeners reported having used FP method because of listening.	Medium

Westoff & Koffman, 2011	Cross-sectional	[Multi-country]	Multi-country	2000 – 2008	Men & women, 15-49 in 48 developing countries	N>0.5 million women	DHS cross-sectional	Regular family planning education programs (Radio, TV, NP)	"Television viewing in particular is strongly associated with reproductive behavior: the more that women watch television, the fewer children they want (including young never-married women as well as married women), the more they use modern contraceptives, and the fewer children they have."	High
Westoff et al., 2011	Cross-sectional	[Multi-country]	Multi-country	2000 – 2008	Men & women, 15-49 in 48 developing countries	N>0.5 million women	DHS cross-sectional	Regular family planning education programs (Radio, TV, NP)	"Television viewing in particular is strongly associated with reproductive behavior: the more that women watch television, the fewer children they want (including young never-married women as well as married women), the more they use modern contraceptives, and the fewer children they have."	High
Westoff, 2012	Cross-sectional	[Multi-country]	Multi-country	2001 – 2012	Women, 15-49 in 52 developing countries	N=787,919	DHS cross-sectional	Regular family planning education programs (Radio, TV, NP)	"General exposure to radio and television shows positive effects in various countries, while media messages promoting family planning seem less important except in West and Middle Africa and in poorer countries elsewhere."	High
Wosu, 2013	Cross-sectional	Lagos Metropolis [Nigeria]	Western Africa	Nov-09 – Feb-10	Men & women in Lagos metropolis	N=1026; multistage sampling	Cross-sectional survey	Regular family planning education programs (Radio, TV, NP)	Positive media sexual health content is likely to promote sexual health among adolescents but negative contents can put adolescents' sexual health in danger	Medium

Figure S1. Possible confounder characteristics explaining various estimated exposure-associated risk ratios (RRs)

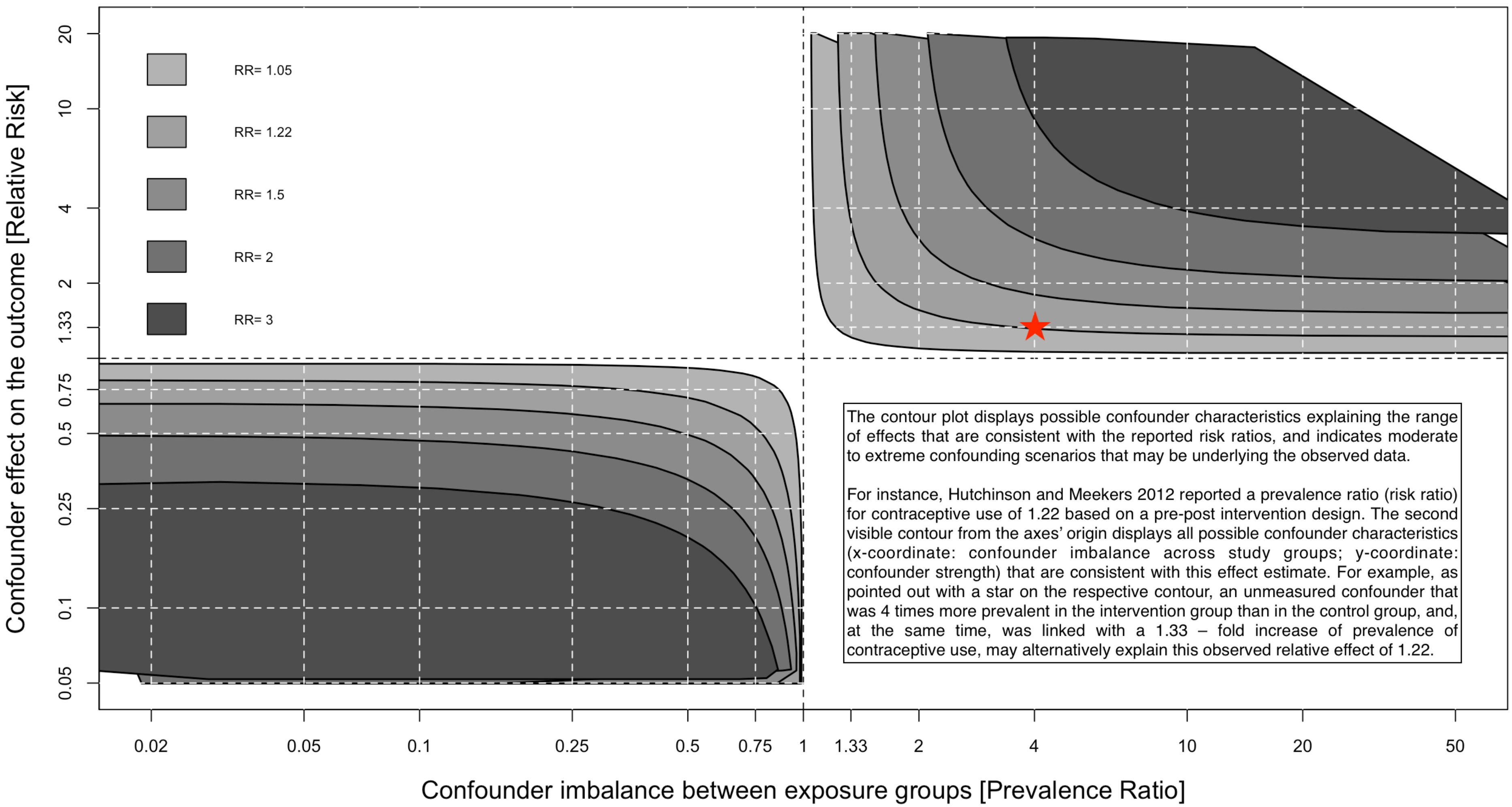

Table S5. Summary of prediction intervals by study design

Design	Outcome	Lower	Upper	Range
Pre-post	Contraceptive knowledge	0.90	1.23	0.33
Longitudinal	Contraceptive knowledge	0.45	2.23	1.78
All	Contraceptive knowledge	0.51	2.36	1.85
Cross-sectional	Contraceptive use	0.10	19.96	19.86
Pre-post	Contraceptive use	0.47	4.13	3.66
Longitudinal	Contraceptive use	0.37	2.74	2.37
All	Contraceptive use	0.55	3.19	2.64

Figure S2a. Association between media programs on reproductive health and contraceptive knowledge by year

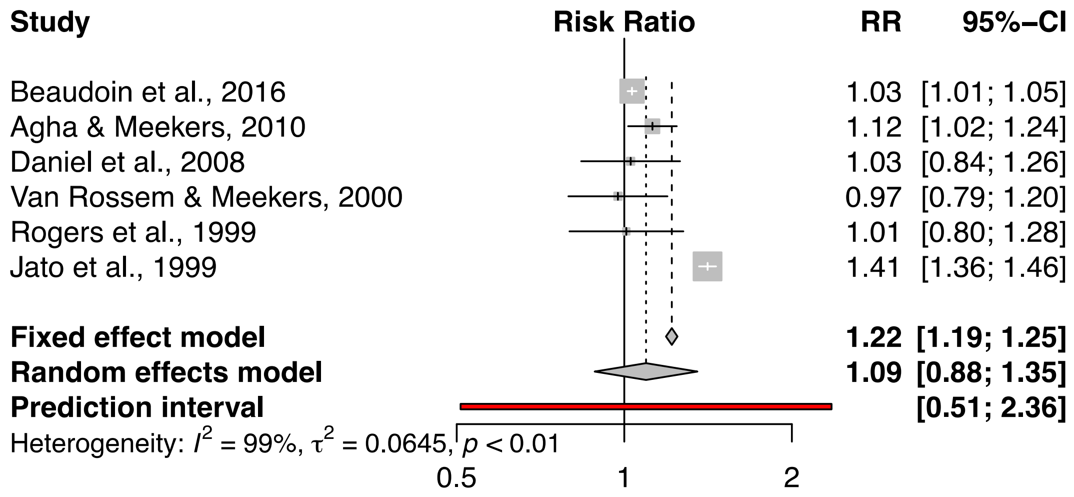

Figure S2b. Association between media programs on reproductive health and contraceptive knowledge by year

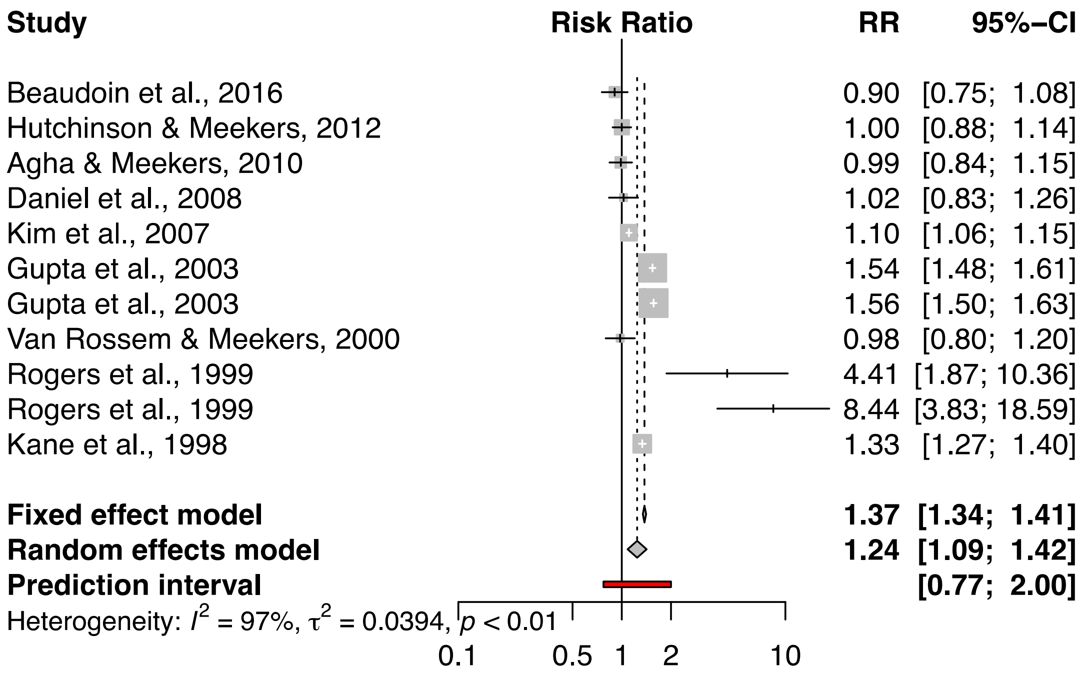

Figure S3. Themes emerging from qualitative coding and thematic analysis

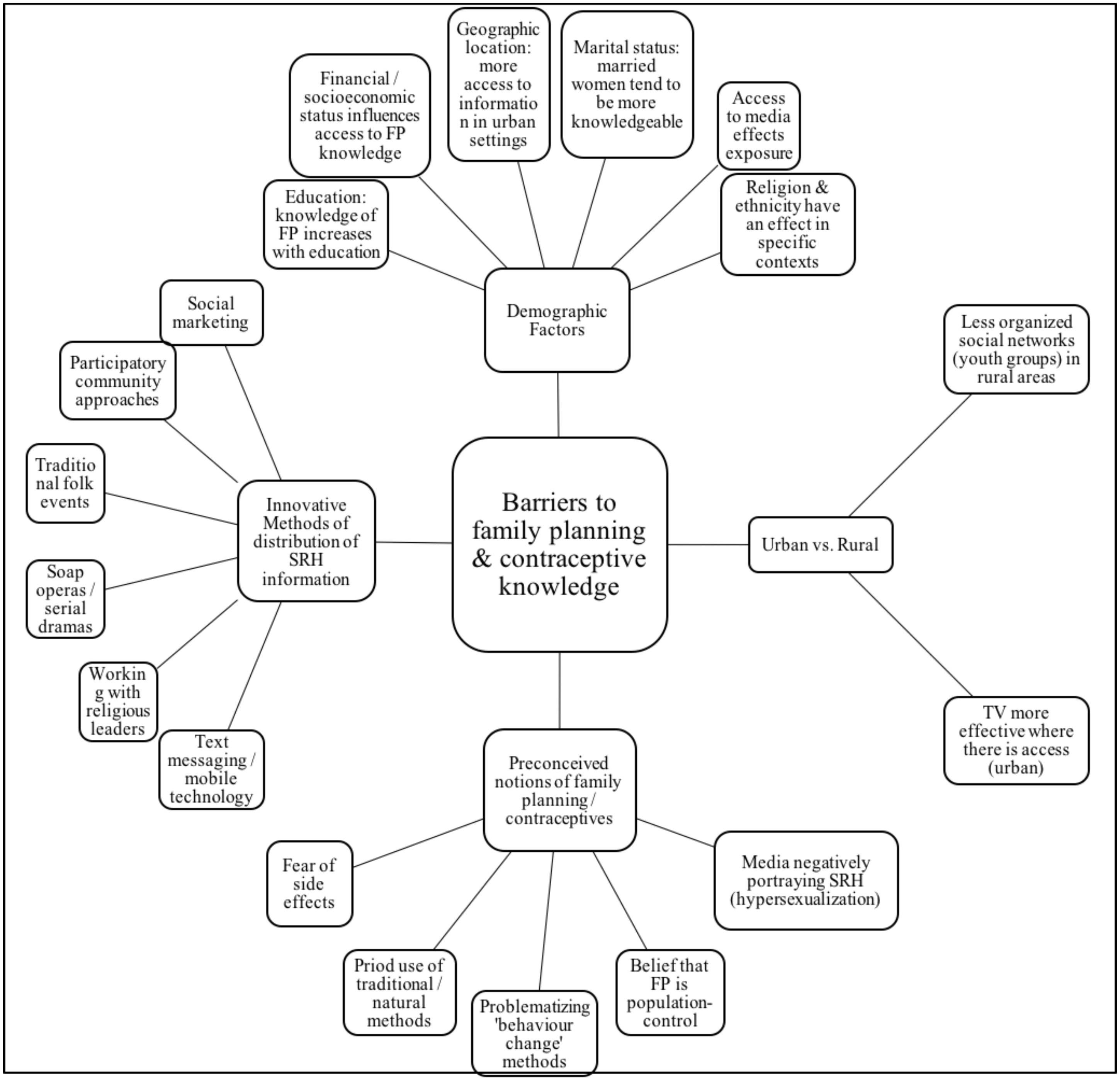

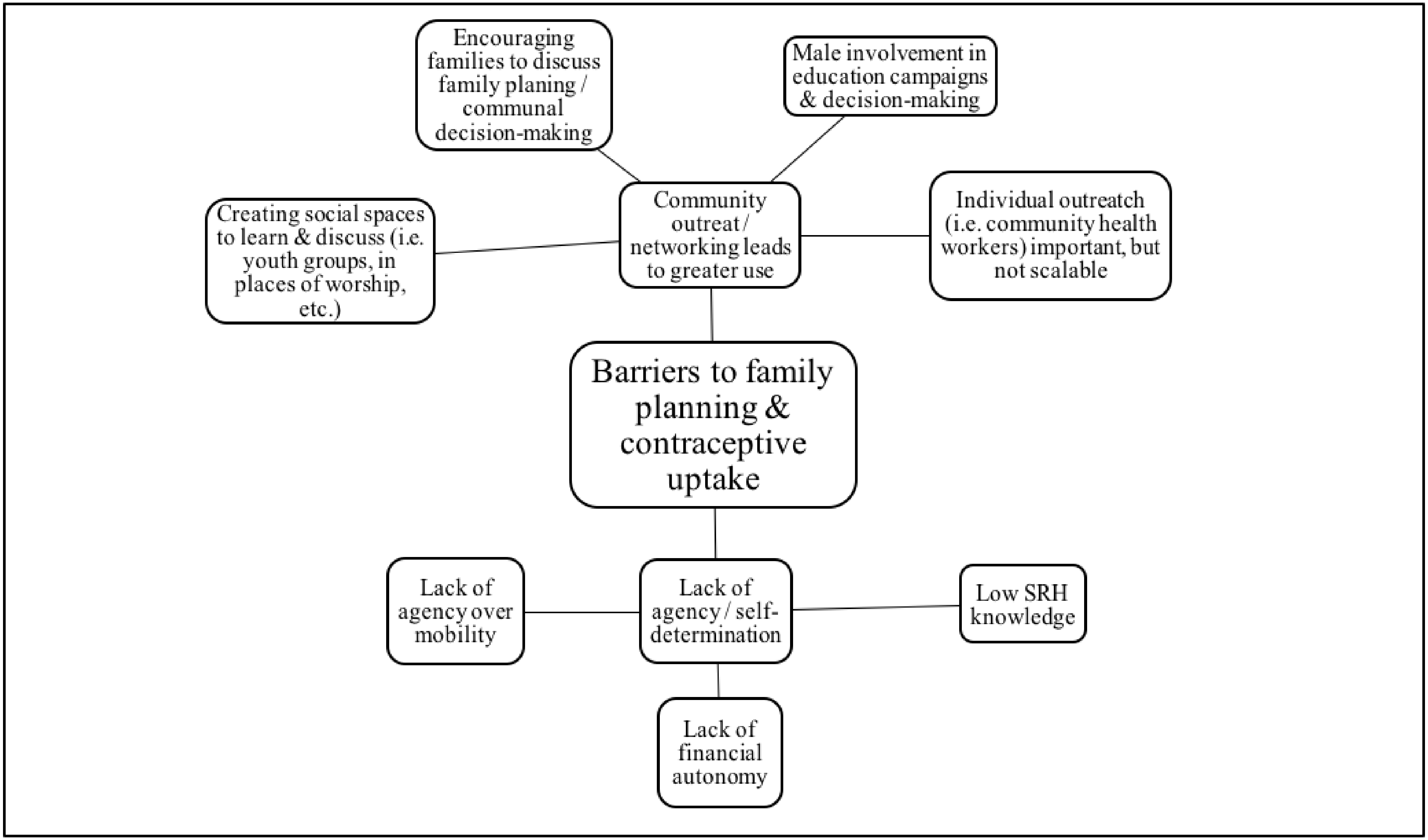
Encouraging families to discuss family planing / communal decision-making
Male involvement in education campaigns & decision-making
Creating social spaces to learn & discuss (i.e. youth groups, in places of worship, etc.)
Community outreat / networking leads to greater use
Individual outreatch (i.e. community health workers) important, but not scalable
Barriers to family planning & contraceptive uptake
Lack of agency over mobility
Lack of agency / self-determination
Low SRH knowledge
Lack of financial autonomy

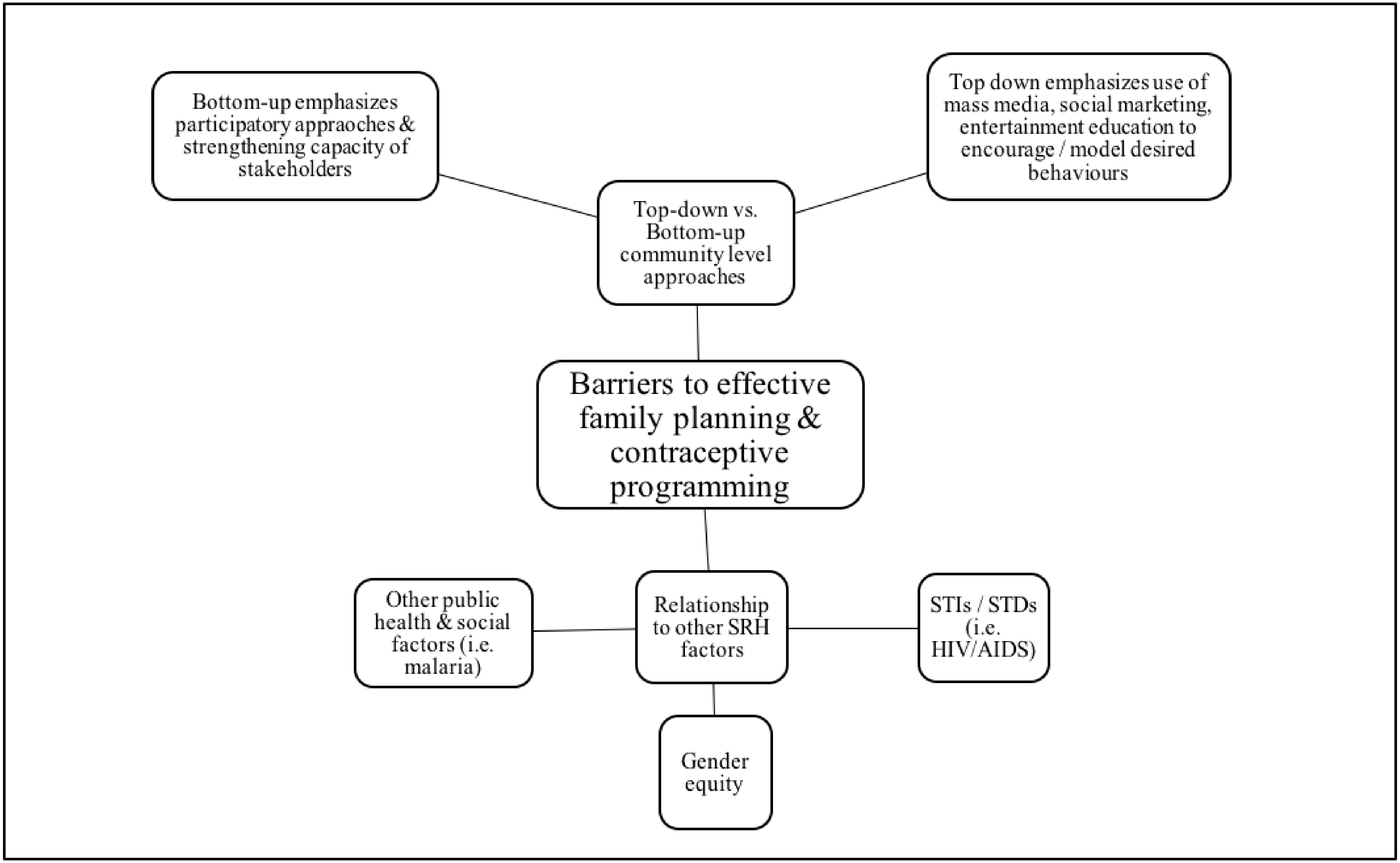
Bottom-up emphasizes participatory appraoches & strengthening capacity of stakeholders
Top down emphasizes use of mass media, social marketing, entertainment education to encourage / model desired behaviours
Top-down vs. Bottom-up community level approaches
Barriers to effective family planning & contraceptive programming
Other public health & social factors (i.e. malaria)
Relationship to other SRH factors
STIs / STDs (i.e. HIV/AIDS)
Gender equity
